# Effect of exogenous stress factors on the biosynthesis of carotenoids and lipids by *Rhodotorula* yeast strains in media containing agro-industrial waste

**DOI:** 10.1007/s11274-019-2732-8

**Published:** 2019-10-01

**Authors:** Anna M. Kot, Stanisław Błażejak, Marek Kieliszek, Iwona Gientka, Joanna Bryś, Lidia Reczek, Katarzyna Pobiega

**Affiliations:** 1 Department of Biotechnology, Microbiology and Food Evaluation, Faculty of Food Sciences, Warsaw University of Life Sciences, Nowoursynowska 159C, 02-776 Warsaw, Poland; 2 Department of Chemistry, Faculty of Food Sciences, Warsaw University of Life Sciences, Nowoursynowska 159C, 02-776 Warsaw, Poland; 3 Department of Civil Engineering, Faculty of Civil and Environmental Engineering, Warsaw University of Life Sciences, Nowoursynowska 159, 02-776 Warsaw, Poland

**Keywords:** Red yeast, SCO, Carotenoids, Osmotic stress, Oxidative stress, Irradiation

## Abstract

In this study, we aimed to determine the effect of exogenous stress factors (sodium chloride as osmotic stressor, hydrogen peroxide as an inducer of oxidative stress, white light irradiation, and low temperature) on the biosynthesis of carotenoids and lipids by red yeast (*Rhodotorula glutinis*, *R. mucilaginosa*, and *R. gracilis*) during cultivation in media containing potato wastewater and glycerol. According to our results, the yeast were able to grow and biosynthesize lipids and carotenoids in the presence of the applied stress factors. Low temperature caused an increase in the biosynthesis of intracellular lipids and carotenoids. *R. gracilis* synthesized lipids (21.1 g/100 g_d.w._) and carotenoids (360.4 µg/g_d.w._) in greater quantities than that of other strains. Under these conditions, there was also an increase in the content of unsaturated fatty acids, especially linoleic and linolenic acids. The highest percentage of polyunsaturated fatty acid (PUFA) (30.4%) was synthesized by the *R. gracilis* yeast after cultivation at 20°C. Their quantity was 2.5-fold greater than that of the biomass grown in control conditions. The contribution of individual carotenoid fractions depended both on the yeast strain and the culture conditions. Induction of osmotic stress and low temperature intensified the biosynthesis of β-carotene (up to 73.9% of the total carotenoid content). In oxidative stress conditions, yeast synthesized torulene (up to 82.2%) more efficiently than under other conditions, whereas white light irradiation increased the production of torularhodin (up to 20.0%).

**Electronic supplementary material:**

The online version of this article (10.1007/s11274-019-2732-8) contains supplementary material, which is available to authorized users.

## Introduction

Yeast belonging to the genus *Rhodotorula* have been described as oleaginous microorganisms. Under specific conditions, these microorganisms can synthesize lipids up to 20% of their dry cellular weight (Kot et al. 2015). They can also biosynthesize carotenoids such as β-carotene, torulene, and torularhodin, which gives them a striking reddish-pink color appearance. Due to this property, they are described as “red yeast” (Kot et al. 2018). The production of lipids and carotenoids with the utilization of microbiological methods show greater advantages than conventional means, such independence from weather conditions, season, geographical location, and availability of agricultural land. Furthermore, microorganisms demonstrate a rapid rate of growth, which significantly shortens the production cycle. However, the only obstacle for a successful production output is the low yield per unit volume of the medium, which greatly increases the cost of the process (Frengova and Beshkova 2009; Kot et al. 2015).

The content of lipids and carotenoids in yeast cells of the genus *Rhodotorula* depends on many factors, such as the source of carbon and nitrogen, presence of microelements in the medium, rate of aeration, and temperature of cultivation (Kot et al. 2016, 2018). However, the biosynthesis of lipids and carotenoids by the yeast cells can be intensified by adding various chemical inducers to the culture medium and by changing the environmental conditions of the culture. These changes are described as stressful conditions, for example, change in temperature, visible or ultraviolet light irradiation, an increase in osmotic pressure (e.g., high concentrations of sodium chloride, sugars, and glycerol), presence of toxic substances (e.g., heavy metal salts, phenol, and methylene blue), and presence of oxidative stress (Sakaki et al. 2001; Bhosale and Gadre 2002; Bhosale 2004; Frengova and Beshkova 2009; Amaretti et al. 2010; Zhang et al. 2014). In response to unfavorable environmental conditions, microbial cells show an increased expression of genes that code for the production of enzymes that are involved in the biosynthesis of various compounds, including lipids and carotenoids (Marova et al. 2012). Due to the presence of these stress-responsive mechanisms, yeasts not only can tolerate higher doses of a given factor after but also other components that can cause stress (Sigler et al. 1999).

Therefore, in this study, we aimed to determine the effect of exogenous stress factors on the biosynthesis of carotenoids and lipids by *Rhodotorula glutinis* LOCKR13, *R. mucilaginosa* ATCC 66034, and *R. gracilis* ATCC 10788 yeast. Among the many stress factors presented in the literature four were selected: sodium chloride as osmotic stressor, hydrogen peroxide as an inducer of oxidative stress, white light irradiation, and low temperature. These factors are easy to apply, cheap, do not cause genetic modification and simultaneously can effectively intensify the biosynthesis of lipids and/or carotenoids by yeasts of the genus *Rhodotorula*. To reduce costs, the culture media were prepared exclusively from two industrial wastes. Glycerol fraction was used as the carbon source, and deproteinized potato wastewater was used as a source of nitrogen and minerals.

## Materials and methods

### Biological material

In this study, we used *R. mucilaginosa* ATCC 66034 and *R. gracilis* ATCC 10788 strains obtained from the American Type Culture Collection and *R. glutinis* LOCKR13 strain obtained from the Collection of Pure Cultures of the Lodz University of Technology in Poland.

### Industrial wastes used as components of media

Glycerol fraction from the production of biodiesel (Bioagra-Oil S.A., Tychy, Poland) was used to prepare the growing media. The content of glycerol was established by a chemical method (Milchert et al. 1995). The content of glycerol was determined by GC–MS (Shimadzu, GCMS-QP2010S) using HP-INNOWax capillary column (30 m × 0.25 mm × 0.25 μm; Industrial Chemistry Research Institute, Warsaw). The content of selected mineral was determined by inductively coupled plasma atomic emission spectroscopy (ICP-AES) (Thermo iCAP 6500) in the Analytical Center of WULS, Warsaw. These analyses are described in detail in our previous article (Kot et al. 2019). Glycerol fraction contained: 60.3% glycerol, 3.3% methanol, 1.1% sodium, 0.024% calcium, 0.007% potassium, 0.004% phosphorus, and 0.003% magnesium and had a pH of 12.1.

Deproteinized potato wastewater was used as the source of nitrogen and minerals, and was prepared in laboratory conditions according to the methodology described in the previous work (Kot et al. 2017). The total nitrogen and protein content were determined using the Kjeldahl method with a conversion factor of 6.25 (Kirk 1950). The content of reducing sugars (per glucose) was measured spectrophotometrically at λ = 550 nm using 3,5-dinitrosalicylic acid (Miller 1959). The chemical oxygen demand index was assessed by the dichromate method using Hach Lange cuvette tests (LCK014) in the Water Centre of the Warsaw University of Life Sciences. Potato wastewater contained 1.98 g/L nitrogen, 12.4 g/L total protein, and 8.3 g/L reducing sugars and was characterized by a high index value of chemical oxygen demand (COD) (29,846 mg O_2_/L).

### Preparation of the inoculum

The yeast inoculum was prepared by inoculating the liquid yeast extract–peptone–dextrose (YPD) medium (20 g/L glucose, 20 g/L peptone, 10 g/L yeast extract, pH 5.0) with the material obtained from the agar slants. The cultures were grown on a reciprocating shaker with a frequency of 200 rpm (SM-30 Control, Edmund Bühler) at 28°C for 24 h. One milliliter of inoculum contained 5 × 10^6^ CFU. Culture media were inoculated with yeast inoculum constituting 10% of the culture volume.

### Cultivation of control cultures

The control cultures were prepared in a medium with deproteinized potato wastewater supplemented with glycerol fraction such that the final concentration of glycerol in the medium was 30 g/L. The final pH of the medium was 5.0 (Kot et al. 2019). Ten milliliters of inoculation cultures were centrifuged (3500×*g*/10 min, Centrifuge 5804R, Eppendorf) and washed twice with sterile demineralized water. The yeast biomass was then suspended in 10 mL of sterile culture medium, mixed thoroughly and added to 90 mL of medium in flat-bottomed flasks. One milliliter of inoculated culture medium contained approximately 5 × 10^5^ CFU. The cultures were grown in 500 mL flat-bottomed flasks (contained 100 mL of medium) on a reciprocating shaker (SM-30 Control, Edmund Bühler) with a frequency of 200 rpm at 28°C for 120 h.

### Cultivation of cultures with exogenous stress factors

To determine the effect of low temperature, the cultures were grown at 20°C. The effect of white light irradiation on the growth of yeast strains was tested using illumination equipment, in which the illuminating element consisted of OSRAM Fluora bulbs (power of 18 W, a beam of light of 550 lm and a wavelength ranging from 400 to 800 nm). To determine the effect of increased osmotic pressure, sodium chloride (50 g/L) was added to the growing medium. Finally, to determine the effect of oxidative stress, the yeast cultures were grown in the presence of 5 mM H_2_O_2_ in the growing media. Tests were performed at 28 °C, except in case of the temperature test.

### Analysis of growth of yeast

To evaluate the growth of yeast in the presence of various stress factors, the yield in the cellular biomass was determined by the gravimetric method. Ten milliliters of culture medium was centrifuged for 10 min at 6000×*g* (Centrifuge 5804R, Eppendorf), and then the biomass was washed twice with sterile deionized water. The wet cell biomass was dried at 105°C until a constant weight was obtained. The results were calculated in grams of dry weight per liter of culture medium (g_d.w._/L).

### Analysis of media composition

In order to analyze of media composition after yeast cultures, the analytical methods described in subsection ‘Industrial wastes used as components’ of media were used.

### Estimation of lipids in yeast biomass and fatty acid profile

The lipid content in yeast cell biomass was determined by the modified Bligh and Dyer method. To lysed the yeast cell wall, the dried biomass was subjected to acid hydrolysis (1 M hydrochloric acid, 60°C, 2 h). Then, the lipids were extracted with a mixture of chloroform and methanol (1:1 v/v), and the samples were centrifuged (3500×*g*/10 min). The lower phase was collected, and the lipid content in the yeast biomass was determined gravimetrically (Zhang et al. 2011). The percentage fraction of fatty acids in the extracted lipids was determined by gas chromatography (TRACE™ 1300, Thermo Scientific, USA) equipped with a flame ionization detector (FID). The fatty acids were esterified with 2 M KOH in methanol. The separation was conducted on an RTX-2330 capillary column (60 m × 0.25 mm × 0.2 μm, Restek, USA). The temperature of the chromatographic oven was set at 50°C (3 min), with a rate of increase in temperature of 3°C/min up to 250°C (5 min). Nitrogen was used as the carrier gas (1.6 mL/min). The injection temperature was set at 230°C and FID was set at 260°C. The identification of fatty acids was performed based on the retention times of Nu-Chek-Prep Inc. standards (USA) present in the GLC 461 mixture (fatty acid esters from C4:0 to C24:0).

### Estimation of carotenoids in yeast biomass and their profile

Determination of the total carotenoid content in yeast biomass was performed by spectrophotometric method. Briefly, the yeast cell wall was lysed with dimethyl sulfoxide (Cutzu et al. 2013). The carotenoids were extracted with a mixture of petroleum ether and acetone (1:1 v/v), and the absorbance of the colored ether layer was measured at λ = 457 nm (UV1800 spectrophotometer, Rayleigh). The total carotenoid content in yeast cell biomass was calculated from the standard curve prepared for the β-carotene standard solutions and was given in microgram per gram of dry matter.

Percentage fractions of β-carotene, torulene, and torularhodin were determined by high-performance liquid chromatography (Agilent 1200 Series, Palo Alto, CA, USA) with a UV–Vis detector (457 nm). Bionacom C18-2 analytical column (250 mm × 4.6 mm, 5 μm) was used for the separation phase. The mobile phase contained a mixture of acetonitrile, isopropanol, and ethyl acetate in a ratio of 4:4:2 by volume, and the flow rate was adjusted to 0.7 mL/min (isocratic). We identified β-carotene based on the retention time of the standard (Sigma-Aldrich). Torulene and torularhodin standards were identified by thin-layer liquid chromatography (TLC) [Kot et al. 2017].

### Statistical analysis

All tests were performed in triplicates. The results were analyzed using the statistical program in the R program (version i386 2.15.3, RCommander tab) specifying the normal distribution of data (Shapiro–Wilk test), homogeneity of variance (Levene test), and significance of differences between averages (one-way analysis of variance and Tukey’s test) at the significance level of α = 0.05. R scripts used for the statistical analysis has been included in the supplementary material (Table S1).

## Results

The present study aimed to increase the intracellular concentration of lipids and carotenoids in the biomass of the *Rhodotorula* yeast strains by enhancing the biosynthesis of these compounds by the application of various stress factors. Based on the results of our previous research [Kot et al. 2019], we selected potato wastewater and 3% glycerol as the basal medium. Under these conditions, *Rhodotorula* yeast proliferated rapidly and metabolized the compounds present in the medium. The cultivation of yeast strains in this medium resulted in greater reduction in COD value and utilization of nitrogen compounds and glycerol when compared to the other tested media. However, the content of intracellular lipids and carotenoids did not exceed 20 g/100 g_d.w._ and 230 µg/g_d.w._, respectively. Therefore, this study was carried out to increase the content of these compounds in the *Rhodotorula* yeast biomass.

In this study, the yeast belonging to the genus *Rhodotorula* showed the ability to grow under oxidative stress, osmotic stress, irradiation, and at low temperature. *Rhodotorula glutinis* yeast was sensitive to the high level of osmotic stress in the culture medium (Figure S1). After 120 h of cultivation, the yield of cellular biomass of *R. glutinis* was two times lower (11.2 g_d.w._/L) than that of *R. mucilaginosa* and *R. gracilis* (20.3 and 22.0 g_d.w._/L, respectively). Exogenous stress factors differentiated the growth of *R. mucilaginosa* yeast within 48 h of cultivation (Figure S2). However, after 120 h of cultivation, the yield of cellular biomass of *R. mucilaginosa* was similar to the 48 h cultivation (21.4 and 21.9 g_d.w._/L, respectively). Among the studied strains, *R. gracilis* was the most sensitive to the applied stress factors. The presence of osmotic stress or oxidative stress inhibited the growth of *R. gracilis* after 24 h of cultivation. A similar effect was observed in cultures after irradiation (Figure S3). Reducing the temperature from 28 to 20 °C during the cultivation increased the rate of growth of *R. gracilis* yeast in the first days of culture. After 120 h of cultivation, the yield of cellular biomass of *R. gracilis* yeast was significantly lower in media containing 5% NaCl (19.0 g_d.w._/L) and 5 mM H_2_O_2_ (15.2 g_d.w._/L). In the remaining cultures biomass yields were from 20.5 to 21.4 g_d.w._/L.

In control and experimental conditions, yeast showed the ability to assimilate glycerol, as well as sugars and nitrogenous compounds present in the potato wastewater. The amount of utilization of these ingredients from the media was dependent on the growth of yeast. Glycerol was utilized by over 96% except cultivation of *R. gracilis* yeast under oxidative stress (61%) and *R. glutinis* under the presence of osmotic stress. In this case the total rate of utilization of glycerol was just over 19% (Table S1). For these variants, the rate of utilization of nitrogenous compounds from the media was significantly lower (32–44%) than that of other (50–66%) compounds. The residue probably contained nitrogen compounds in the form that the yeast strains were not able to assimilable or was composed of secondary yeast metabolites such as ammonia. The reducing sugars present in the potato wastewater were effectively utilized by yeast (up to 87%). The remaining sugars could be galactose and rhamnose, which are used as carbon sources only by some of the strains belonging to the genus *Rhodotorula* [Fell and Statzell-Tallman 1998; Kot et al. 2019].

The ability of *Rhodotorula* yeast to biodegrade deproteinized potato wastewater and glycerol fraction was determined by measuring the COD index of the culture media. According to the results (Table S1), both types of industrial wastes were partially utilized by yeasts. After 120 h of cultivation, the COD values of the culture media decreased by 70–83%, which amounted to 10,873–18,880 mg O_2_/L. The only exceptions were *R. glutinis* yeast cultures grown under osmotic stress (42,530 mg O_2_/L) and *R. gracilis* cultures grown under oxidative stress conditions (24,273 mg O_2_/L). This was due to a significant reduction in glycerol metabolism under these conditions. As in our previous studies (Kot et al. 2019), it was not possible to reduce the COD value to a level that would enable the utilization of these post-culture media in natural conditions in accordance with the requirements of the Regulation of the Minister of the Environment from (2014).

In this study, the *Rhodotorula* yeast showed the ability to biosynthesize intracellular lipids under control and experimental conditions. Reducing the temperature of cultivation to 20°C intensified the biosynthesis of lipids by *R. glutinis* yeast (Table 1). After 120 h of cultivation at 20°C, the lipid content produced by *R. glutinis* increased by 67% (17.4 g/100 g_d.w._) compared to the control culture (10.4 g/100 g_d.w._). The biomass of *R. mucilaginosa* obtained after cultivation in control and experimental conditions (osmotic stress, oxidative stress, and under white light irradiation) showed similar yield in of intracellular lipids, which after the 120 h ranged from 10.9 to 13.4 g/100 g_d.w._. Similar to *R. glutinis*, low temperature (20°C) intensified the process of lipid biosynthesis by *R.mucilaginosa* and its content (16.3 g/100 g_d.w._) increased by 31% compared to the control conditions (12.4 g/100 g_d.w._). Among all the studied strains, *R. gracilis* synthesized the greatest amounts of intracellular lipids. After 120 h of cultivation at low temperature, the total content of cellular lipid was 21.1 g/100 g_d.w._; therefore, under these tested conditions, *R. gracilis* yeast fulfilled the criterion of being in the category of oleaginous yeast. It synthesized 36% higher amounts of lipids than that of the control culture (15.5 g/100 g_d.w._). In the remaining experimental conditions (under osmotic stress, oxidative stress, and white light irradiation), the lipid content obtained after 120 h of cultivation was similar (14.3–14.9 g/100 g_d.w._) to that obtained under control conditions. The highest value of the volumetric yield of lipid biosynthesis (4.52 g/L) and productivity (0.038 g/L/h) was obtained after 120 h of *R. gracilis* yeast cultivation at 20 °C. For *R. glutinis* and *R. mucilaginosa* yeasts values of Y_L_ and Q_L_ also were the highest and were respectively 3.82 g/L and 0.032 g/L/h for *R. glutinis* and 3.49 g/L and 0.029 g/L/h for *R. mucilaginosa*. At 20°C, the highest yield of lipids per consumed carbon source was obtained for *R. gracilis* yeast (0.124 g/g), which confirms the suitability of *R. gracilis* strain for the synthesis of lipids from waste materials.

**Table 1:** Parameters characterizing the biosynthesis of lipids by *Rhodotorula* yeast strains after 120 h of cultivation in control and experimental conditions

Culture conditions	Total lipid content in yeast biomass (g/100 g_d.w._)	Y_L_ (g/L)	Q_L_ (g/L/h)	Y_L/CS_ (g/g)
*Rhodotorula glutinis*				
Control	10.4 ± 0.4^b^	2.11 ± 0.07^b^	0.018 ± 0.001^b^	0.057 ± 0.002^b^
+ 5% NaCl	12.4 ± 1.5^b^	1.40 ± 0.30^c^	0.012 ± 0.002^c^	0.111 ± 0.032^a^
+ 5 mM H_2_O_2_	10.9 ± 0.6^b^	2.25 ± 0.21^b^	0.019 ± 0.002^b^	0.060 ± 0.005^b^
Culture at 20°C	17.4 ± 1.4^a^	3.82 ± 0.32^a^	0.032 ± 0.003^a^	0.103 ± 0.009^a^
White light irradiation	10.7 ± 0.4^b^	2.26 ± 0.14^b^	0.019 ± 0.001^b^	0.062 ± 0.004^b^
*Rhodotorula mucilaginosa*				
Control	12.4 ± 1.5^b^	2.71 ± 0.30^b^	0.023 ± 0.003^b^	0.075 ± 0.008^b^
+ 5% NaCl	13.3 ± 1.9^b^	2.87 ± 0.38^b^	0.024 ± 0.003^b^	0.080 ± 0.011^ab^
+ 5 mM H_2_O_2_	12.5 ± 0.7^b^	2.71 ± 0.06^b^	0.023 ± 0.001^b^	0.073 ± 0.003^b^
Culture at 20°C	16.3 ± 1.4^a^	3.49 ± 0.24^a^	0.029 ± 0.002^a^	0.095 ± 0.011^a^
White light irradiation	11.0 ± 0.9^b^	2.34 ± 0.21^c^	0.020 ± 0.002^c^	0.064 ± 0.004^c^
*Rhodotorula gracilis*				
Control	15.5 ± 1.2^b^	3.30 ± 0.30^b^	0.027 ± 0.002^b^	0.089 ± 0.010^b^
+ 5% NaCl	14.4 ± 1.8^b^	2.73 ± 0.37^c^	0.023 ± 0.003^b^	0.084 ± 0.008^b^
+ 5 mM H_2_O_2_	14.3 ± 0.8^b^	2.17 ± 0.05^d^	0.018 ± 0.000^c^	0.084 ± 0.004^b^
Culture at 20°C	21.1 ± 1.11^a^	4.52 ± 0.36^a^	0.038 ± 0.003^a^	0.124 ± 0.007^a^
White light irradiation	14.8 ± 2.0^b^	3.05 ± 0.55^bc^	0.025 ± 0.005^b^	0.082 ± 0.013^b^

Y_L_—volumetric yield of lipid (grams per liter of medium); Q_L_—volumetric lipid productivity (grams per liter of medium per hour); Y_L/CS_—yield of lipids per consumed carbon source (gram of lipid per gram of consumed carbon sources); a, b, c…—indexes mean homogeneous groups determined by Tukey’s test at the level of significance 0.05

**Fig. 1:**
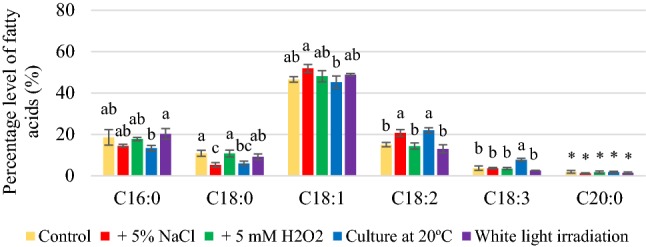
Percentage levels of fatty acids synthesized by *Rhodotorula glutinis* yeast after 120 h of cultivation in control and experimental conditions (a, b, c…—indexes mean homogeneous groups, *no significant differences, Tukey’s test, α = 0.05)

**Fig. 2:**
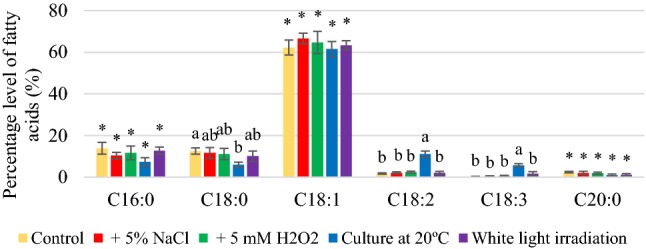
Percentage levels of fatty acids synthesized by *Rhodotorula mucilaginosa* yeast after 120 h of cultivation in control and experimental conditions (a, b, c…—indexes mean homogeneous groups, *no significant differences, Tukey’s test, α = 0.05)

**Fig. 3:**
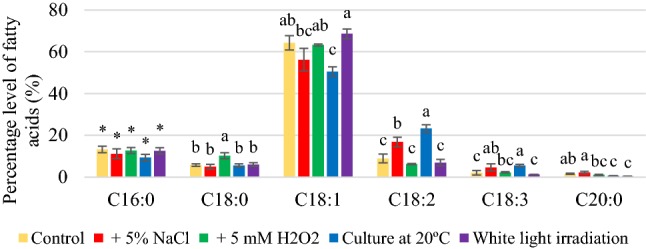
Percentage levels of fatty acids synthesized by *Rhodotorula gracilis* yeast after 120 h of cultivation in control and experimental conditions (a, b, c…—indexes mean homogeneous groups, *no significant differences, Tukey’s test, α = 0.05)

Depending on the culture conditions, fatty acid composition was dominated by palmitic, stearic, oleic, and linoleic, and their percentages were dependent on the culture conditions (Figs. 1, 2, 3). The yeast synthesized oleic acid (45.3–68.5%). Reducing the temperature of growing conditions from 28 to 20 °C significantly increased the content of unsaturated fatty acids (Figs. S4, S5, S6), especially linoleic and linolenic acids. The percentage of linolenic acid was respectively 22.0 and 23.2% obtained for *R. glutinis* and *R. gracilis* yeasts. However, *R. mucilaginosa* synthesized linolenic acid in a significantly less quantity than the other two strains (11.1%), but it was six-fold greater than that of the control culture (1.8%). The highest amount of PUFA (30.4%) was found in lipids extracted from *R. gracilis* yeast biomass after cultivation at 20 °C, which was 2.5 times more than that of the control culture (Figure S6). This was due to the presence of increased amounts of linoleic and linolenic acids. *R. glutinis* yeast also efficiently synthesized linoleic and linolenic acids at 20 °C, and the amount of PUFA was determined at around 29.7% (Fig. S4). For *R. glutinis* and *R. gracilis* yeast strains, a significant increase in the amount of linoleic and linolenic acids was also found in the medium representing osmotic stress; however, the quantity was lower than that of cultivation at 20 °C. Thus, the yeast biomass of *R. glutinis* and *R. gracilis* strains obtained after cultivation at 20°C in the media containing potato wastewater supplemented with 3% glycerol can be recommended as an additive to livestock feed supplement containing unsaturated fatty acids, especially oleic, linoleic, and linolenic acid.

The amount of carotenoids synthesized by yeast strains was determined under the conditions of exogenous stress (Table 2). After 120 h of cultivation under osmotic stress, oxidative stress, and white light irradiation, the amount of carotenoid biosynthesis in the yeast biomass of *R. glutinis* was similar in both control and experimental conditions (ranging from 220.7 to 249.1 µg/g_d.w._). Carotenoid biosynthesis was found to be increased after cultivation at 20°C (280.3 µg/g_d.w._). The same relationship was found for *R. mucilaginosa* yeast. After 120 h of cultivation at 20°C, *R. mucilaginosa* synthesized 46% more total carotenoids (150.7 µg/g_d.w._) than that of the control culture (103.0 µg/g_d.w._). The process of carotenoid biosynthesis by *R. gracilis* yeast was increased under low temperatures (20 °C) and white light irradiation. After 120 h of incubation, *R. gracilis* yeast synthesized 57% (360.4 µg/g_d.w._) and 34% (307.2 µg/g_d.w._) more carotenoids than that of control culture (229.9 µg/g_d.w._). According to our results, low temperature most effectively stimulated the biosynthesis of carotenoid by *Rhodotorula* yeasts. After taking the yield of cellular biomass into account, the highest volumetric yield of carotenoid biosynthesis (7.72 mg/L) was found after cultivation of *R. gracilis* at 20°C, which was around 57% increase in the volumetric yield). Most of the carbon was utilized for the biosynthesis of carotenoids by *R. gracilis* during cultivation at 20°C. The highest yield of carotenoids per consumed carbon source was 0.212 mg/g, and the strains utilized more than 60% of the carbon for the biosynthesis of carotenoids than that of control conditions (0.132 mg/g).

**Table 2:** Parameters characterizing the biosynthesis of carotenoids by *Rhodotorula* yeast after 120 h of cultivation in control and experimental conditions

Culture conditions	Total carotenoid content in biomass (µg/g_d.w._)	Y_CAR_ (mg/L)	Q_CAR_ (mg/L/h)	Y_CAR/CS_ (mg/g)
*Rhodotorula glutinis*				
Control	235.6 ± 18.9^b^	4.78 ± 0.48^b^	0.040 ± 0.004^b^	0.130 ± 0.012^c^
+ 5% NaCl	249.1 ± 12.1^ab^	2.79 ± 0.26^c^	0.023 ± 0.002^c^	0.220 ± 0.038^a^
+ 5 mM H_2_O_2_	228.2 ± 16.3^b^	4.69 ± 0.15^b^	0.039 ± 0.001^b^	0.126 ± 0.005^c^
Culture at 20 °C	280.3 ± 16.0^a^	6.16 ± 0.50^a^	0.051 ± 0.004^a^	0.167 ± 0.014^b^
White light irradiation	220.7 ± 14.7^b^	4.67 ± 0.43^b^	0.039 ± 0.004^b^	0.129 ± 0.012^c^
*Rhodotorula mucilaginosa*				
Control	103.0 ± 10.5^b^	2.26 ± 0.26^b^	0.019 ± 0.002^b^	0.062 ± 0.006^b^
+ 5% NaCl	111.2 ± 9.3^b^	2.40 ± 0.15^b^	0.020 ± 0.001^b^	0.067 ± 0.006^b^
+ 5 mM H_2_O_2_	115.5 ± 12.9^b^	2.50 ± 0.35^b^	0.021 ± 0.003^b^	0.068 ± 0.010^b^
Culture at 20 °C	150.7 ± 11.8^a^	3.23 ± 0.29^a^	0.027 ± 0.002^a^	0.088 ± 0.009^a^
White light irradiation	108.4 ± 10.8^b^	2.32 ± 0.27^b^	0.019 ± 0.002^b^	0.063 ± 0.008^b^
*Rhodotorula gracilis*				
Control	229.9 ± 18.7^c^	4.89 ± 0.49 ^cd^	0.041 ± 0.004^c^	0.132 ± 0.018^c^
+ 5% NaCl	211.5 ± 15.7^c^	4.00 ± 0.24^d^	0.033 ± 0.002^d^	0.123 ± 0.011^c^
+ 5 mM H_2_O_2_	277.1 ± 17.1^b^	4.22 ± 0.53^d^	0.035 ± 0.004^d^	0.163 ± 0.013^b^
Culture at 20 °C	360.4 ± 11.1^a^	7.72 ± 0.28^a^	0.064 ± 0.002^a^	0.212 ± 0.006^a^
White light irradiation	307.2 ± 7.9^b^	6.29 ± 0.36^b^	0.052 ± 0.003^b^	0.170 ± 0.007^b^

Y_CAR_—volumetric yield of carotenoids (milligrams per liter of medium); Q_CAR_—volumetric carotenoids productivity (milligrams per liter of medium per hour); Y_CAR/CS_—yield of carotenoids per consumed carbon source (milligram of carotenoids per gram of consumed carbon sources); a, b, c…—indexes mean homogeneous groups determined by Tukey’s test at the level of significance 0.05

The individual fractions of carotenoid depended both on the yeast strain and on the culture conditions (Table 3). Osmotic stress stimulated the biosynthesis of β-carotene by *R. glutinis* yeast. After 120 h of cultivation, the amount of β-carotene was 50.3% of the total carotenoid fraction, whereas in the control culture, it was 10% lower than the experimental culture. The same pattern was observed for the culture at 20°C, and the proportion of β-carotene synthesized by *R. glutinis* under these conditions was significantly higher (62.7%) than that of the other strains. The presence of oxidative stress was found to stimulate the biosynthesis of torulene (76.8%). Irradiation with white light stimulated the production of torularhodin (21.5%). In the control and experimental conditions, the *R. mucilaginosa* yeast primarily synthesized torulene and β-carotene. The percentage of torulene was the highest (82.2%) after cultivation in of yeast cells under oxidative stress. Similar to *R. glutinis* yeast, there was a significant increase (more than 50%) in the synthesis of β-carotene by *R. mucilaginosa* yeast under osmotic stress and at 20°C. Irradiation with white light increased the production of torularhodin by *R. mucilaginosa*. In comparison to the control culture (3.8%), *R. mucilaginosa* grown under irradiation showed a four-fold (16.4%) increased the production of torularhodin. Irradiation of *R. gracilis* culture with white light resulted in a significant increase in the percentage of torulene (76.1%). Similar to the other two strains, *R. gracilis* yeast synthesized torulene in greater quantities under oxidative stress, whereas osmotic stress and low temperature increased the production of β-carotene.

**Table 3:** The percentage levels of β-carotene, torulene, and torularhodin in carotenoid fractions extracted from *Rhodotorula* yeast biomass after 120 h of cultivation

Culture conditions	β-carotene	Torulene	Torularhodin
*Rhodotorula glutinis*			
Control	38.4 ± 1.8^c^	48.9 ± 7.8^b^	7.8 ± 2.7^b^
+ 5% NaCl	50.3 ± 3.4^b^	44.0 ± 3.7^b^	3.4 ± 1.1^c^
+ 5 mM H_2_O_2_	20.4 ± 3.8^d^	76.8 ± 3.5^a^	1.4 ± 0.7^d^
Culture at 20 °C	62.7 ± 2.2^a^	33.6 ± 2.4^c^	0.4 ± 0.3^e^
White light irradiation	40.7 ± 1.8^c^	35.4 ± 2.2^c^	21.5 ± 1.6^a^
*Rhodotorula mucilaginosa*			
Control	18.2 ± 1.7^b^	74.8 ± 0.7^b^	3.8 ± 1.2^b^
+ 5% NaCl	38.8 ± 1.7^a^	56.4 ± 3.4^d^	0.8 ± 0.4 ^cd^
+ 5 mM H_2_O_2_	15.1 ± 3.0^bc^	82.2 ± 3.6^a^	1.6 ± 0.9^c^
Culture at 20 °C	45.4 ± 5.9^a^	53.1 ± 6.0^d^	0.5 ± 0.2^d^
White light irradiation	12.9 ± 2.0^c^	67.8 ± 2.2^c^	16.4 ± 1.0^a^
*Rhodotorula gracilis*			
Control	59.5 ± 3.0^c^	38.8 ± 2.3^c^	nd
+ 5% NaCl	75.5 ± 2.5^a^	22.5 ± 2.1^e^	nd
+ 5 mM H_2_O_2_	32.8 ± 2.7^d^	65.6 ± 3.0^b^	nd
Culture at 20 °C	68.2 ± 2.9^b^	30.7 ± 2.5^d^	nd
White light irradiation	22.0 ± 2.0^e^	76.1 ± 2.0^a^	nd

*Nd* not detected

a, b, c…—indexes mean homogeneous groups determined by Tukey’s test at the level of significance 0.05

## Discussion

According to the results of this study, the applied exogenous stress factors stimulated the biosynthesis of various compounds by the yeast in the presence of agro-industrial waste. The low temperature increased the amount of biosynthesis and accumulation of intracellular lipids. It is generally recognized that the optimum temperature for the growth and biosynthesis of lipids by oleaginous yeasts is 30 °C (Mohammed et al. 2018); however, some strains synthesize lipids more efficiently at lower temperatures (Zhang et al. 2014), which may result from the higher expression of genes coding for the production of specific desaturases (He et al. 2015). Modification of the composition of lipids is a natural mechanism of adaptation of microorganisms to low temperatures, ensuring the fluidity of plasma membranes, which determines the proper course of cellular transport processes (Suutari et al. 1990; He et al. 2015).

Based on the results of this study, low temperature (20°C) effectively increased the biosynthesis of carotenoids by the yeast strains. A similar relationship was also observed by Martin et al. (1993), Vijayalakshmi et al. (2001), Bhosale and Gadre (2002), and Nasrabadi and Razavi (Nasrabadi and Razavi 2011). Vijayalakshmi et al. (2001) decreased the incubation temperature of *R. gracilis* yeast from 32 to 24°C which resulted in an increase in the content of carotenoids from 148 to 622 µg/100 g_d.w._. The incubation temperature influences the process of carotenogenesis by regulating the concentration and activity of enzymes that catalyze the reactions taking place in this pathway (Hayman et al. 1974; Frengova et al. 1995); however, its effect is dependent on the strain, composition of the medium, and other environmental parameters.

The applied exogenous stress factors significantly affected the profile of carotenoids synthesized by the yeast strains, such as torularhodin, torulene, and β-carotene. The biosynthesis of torularhodin was stimulated after the irradiation of yeast cells with white light. These results agree with those reported by Sakaki et al. (2001). They found that increased biosynthesis of torularhodin is an important protective mechanism of yeast cells against radiation damage because it has the ability to neutralize free radicals, especially singlet oxygen. Although singlet oxygen molecules and other free radicals are mainly generated during exposure to UV radiation (Moliné et al. 2009), it cannot be ruled out that illumination of cells in the visible range increased the production of free radicals in yeast cells.

In this study, the addition of hydrogen peroxide to the culture medium led to the increased biosynthesis of torulene by all the yeast strains. Irazusta et al. (2013) also reported that oxidative stress (5 mM H_2_O_2_) stimulated the biosynthesis of torulene by *R. mucilaginosa* RCL-11 yeast; the fraction of torulene in the total pool of carotenoids synthesized was obtained from 21.3 to 45.6%. Thus, it is safe to assume that under in vivo conditions, torulene exhibits higher activity against reactive oxygen species than that of β-carotene and torularhodin (Li et al. 2017). The ability to neutralize the harmful effects of hydrogen peroxide by torulene and torularhodin recently has been confirmed by Du et al. (2017).

Low temperature and osmotic stress stimulated the biosynthesis of β-carotene by *Rhodotorula* yeast. This phenomenon was also observed by Simpson et al. (1964), Frengova et al. (1995), Bhosale and Gadre (2002), and Cheng and Yang (2016). Bhosale and Gadre (2002) found that decreasing the temperature of cultivation of *R. glutinis* yeast from 32 to 20 °C, increased the production of β-carotene from 66 to 92%, as well increased the total carotenoids content from 125.0 to 250 mg/L. The temperature of the culture medium during cultivation of yeast biomass affects the level of enzymes involved in the biosynthetic pathway of these compounds. At low temperatures, enzymes involved in the conversion of γ-carotene to torulene are less active, as a result of which γ-carotene is converted mainly to β-carotene [Simpson et al. 1964; Bhosale 2004].

In this study, osmotic stress stimulated the biosynthesis of β-carotene by all the yeast strains. This result was also obtained by Bhosale and Gadre (2001) during cultivation of *R. glutinis* no. 32 yeast in a medium based on seawater, which contained 23.1 g/L of sodium chloride. Under these conditions, a two-fold increase in the content of β-carotene and a simultaneous reduction (twofold) in the yield of torulene was observed in relation to the control culture medium prepared with distilled water. According to the authors, there was higher activity of lycopene cyclase, which was stimulated by the presence of micronutrients in seawater.

The analysis of our own results and those reported by other authors indicated that the tested stress factors can effectively enhance the biosynthesis of carotenoids and lipids by the red yeast. The degree of intensification and trend of changes in the profile of carotenoids and fatty acids depended on the type of stress factor used, the strain of red yeast used, the composition of the basic culture medium, and the culture conditions. The main similarities between the our results and those reported by other authors relate to: a) an increase in the pool of unsaturated fatty acids during culturing at low temperature; b) an increase in the content of carotenoids in the yeast biomass after cultivation at a low temperature, and c) changes in the levels of individual carotenoid fractions (β-carotene, torulene, torularhodin) depending on the type of stress factor applied. A significant difference was observed with regard to the optimum temperature required for the lipid biosynthesis. In this study, a relevant increase in lipid content was demonstrated after cultivation at 20 °C, while other studies showed that the highest content of these compounds in biomass was obtained for cultures incubated at a temperature of about 30 °C. The results show that the optimal culture temperature should be determined for each yeast strain, which in turn depends on the culture medium used. The composition of the experimental medium could also have contributed to the obtained different results. It is probable that lipid biosynthesis would occur more efficiently at higher temperatures when a different media is used. It is also possible that the type of medium used had a strong impact on the degree of escalation of lipid and carotenoid biosynthesis in the other conditions studied; however, in order to verify this hypothesis, tests should be carried out using at least several different types of basal media for comparison.

## Conclusion

This study shows the possibility of modifying the content and profile of lipids and carotenoids that are synthesized by yeast belonging to the genus *Rhodotorula* by modifying the culture conditions (e.g., under osmotic and oxidative stress, irradiation with white light, and by lowering the temperature). Among the tested exogenous stress factors, the biosynthesis of intracellular lipids, as well as carotenoids, was most effective at 20 °C. At 20 °C, *R. gracilis* synthesized 21.1 g/100 g_d.w._ lipids, which also significantly increased the content of unsaturated fatty acids in yeast lipids, especially linoleic and linolenic acids. In addition, low temperature stimulated the biosynthesis of carotenoids by all the yeast strains, and their highest content (360.4 µg/g_d.w._) was recorded for *R. gracilis* yeast after 120 f of cultivation. The applied stress factors significantly affected the profile of carotenoids synthesized by the yeasts. Furthermore, we found a significant increase in the proportion of β-carotene in the total pool of synthesized carotenoids at low temperatures and under osmotic stress. Under oxidative stress, the yeast strains synthesized more torulene than that of the control conditions. In addition, irradiation stimulated the biosynthesis of torularhodin (*R. glutinis* and *R. mucilaginosa*) and torulene (*R. gracilis*), which shows that these chemicals are an important protective factor of cells against the oxidative stress.

## Electronic supplementary material

Below is the link to the electronic supplementary material.

Supplementary material 1 (DOCX 54 kb)
