# Supplementary material for: Effect of exogenous stress factors on the biosynthesis of carotenoids and lipids by Rhodotorula yeast strains in media containing agro-industrial waste
Source: World J Microbiol Biotechnol. 2019 Oct 1;35(10):157. doi: 10.1007/s11274-019-2732-8 (PMC6773817; doi:10.1007/s11274-019-2732-8)
Supplement: Supplementary file 1 — Supplementary material 1 (DOCX 54 kb) [file 11274_2019_2732_MOESM1_ESM.docx]

**­Supplementary Material**

**Effect of exogenous stress factors on the biosynthesis of carotenoids and lipids by *Rhodotorula* yeast strains in media containing agro-industrial waste**

Anna M. Kot^1*^, Stanisław Błażejak^1^, Marek Kieliszek^1^, Iwona Gientka^1^, Joanna Bryś^2^, Lidia Reczek^3^, Katarzyna Pobiega^1^

^1^Department of Biotechnology, Microbiology and Food Evaluation, Faculty of Food Sciences, Warsaw University of Life Sciences, Nowoursynowska 159C, 02–776 Warsaw

^2^Department of Chemistry, Faculty of Food Sciences, Warsaw University of Life Sciences, Nowoursynowska 159C, 02–776 Warsaw

^3^Department of Civil Engineering, Faculty of Civil and Environmental Engineering, Warsaw University of Life Sciences, Nowoursynowska 159, 02–776 Warsaw

*Corresponding author: Anna M. Kot, E-mail: [anna_kot@sggw.pl](mailto:anna_kot@sggw.pl)

**Contact for other Authors:**

Stanisław Błażejak: [stanislaw_blazejak@sggw.pl](mailto:stanislaw_blazejak@sggw.pl)

Marek Kieliszek: [marek_kieliszek@sggw.pl](mailto:marek_kieliszek@sggw.pl)

Iwona Gientka: [iwona_gientka@sggw.pl](mailto:iwona_gientka@sggw.pl)

Joanna Bryś: [joanna_brys@sggw.pl](mailto:joanna_brys@sggw.pl)

Lidia Reczek: [lidia_reczek@sggw.pl](mailto:lidia_reczek@sggw.pl)

Katarzyna Pobiega: [katarzyna_pobiega@sggw.pl](mailto:katarzyna_pobiega@sggw.pl)

**Table S1.** R scripts used for the statistical analysis

| Type of analysis | R script |
| --- | --- |
| Shapiro–Wilk test | normalityTest(~Value, test="shapiro.test", data=Dataset) |
| Levene test | leveneTest(Value ~ TypeofMedium, data=Dataset, center="median") |
| Analysis of variance | Anova(LinearModel.1, type="II") |
| Tukey’s test | HSD.test(LinearModel.2, " TypeofMedium ", group=T, console=T) |

********Interpretation: Value – column with data; TypeofMedium – column with medium types*

**Table S2.** The percentage usage of glycerol, reducing sugars, and nitrogen from the media and the reduction in the index value of chemical oxygen demand after 120 h of cultivation of *Rhodotorula* yeast strains

| Culture conditions | Percentage utilization of glycerol  [%] | Percentage utilization of reducing sugars  [%] | Percentage utilization of  nitrogen [%] | Percentage reduction  of COD index value  [%] |
| --- | --- | --- | --- | --- |
| *Rhodotorula glutinis* | | | | |
| Control | 98.3 ± 1.0 | 83.4 ± 1.3 | 62.6 ± 7.6 | 75.8 ± 5.0 |
| +5% NaCl | 19.6 ± 2.6 | 86.9 ± 2.7 | 32.0 ± 5.8 | 33.2 ± 5.0 |
| +5 mM H_2_O_2_ | 97.7 ± 1.5 | 82.8 ± 1.9 | 64.6 ± 3.9 | 79.6 ± 4.4 |
| Culture at 20°C | 97.3 ± 2.4 | 84.7 ± 6.9 | 66.2 ± 7.7 | 77.5 ± 3.8 |
| White light irradiation | 96.8 ± 1.4 | 83.9 ± 4.3 | 61.8 ± 4.6 | 74.4 ± 4.5 |
| *Rhodotorula mucilaginosa* | | | | |
| Control | 98.0 ± 1.4 | 84.7 ± 3.9 | 63.0 ± 2.8 | 78.8 ± 2.7 |
| +5% NaCl | 98.1 ± 1.7 | 83.2 ± 1.9 | 65.8 ± 3.4 | 80.3 ± 4.7 |
| + 5 mM H_2_O_2_ | 98.8 ± 0.8 | 79.4 ± 7.7 | 60.7 ± 4.1 | 77.8 ± 4.5 |
| Culture at 20°C | 98.9 ± 0.6 | 81.9 ± 4.8 | 60.2 ± 2.9 | 83.2 ± 3.5 |
| White light irradiation | 96.8 ± 1.6 | 84.5 ± 1.6 | 61.1 ± 2.7 | 81.4 ± 2.2 |
| *Rhodotorula gracilis* | | | | |
| Control | 98.4 ± 1.5 | 81.5 ± 2.1 | 63.1 ± 5.9 | 78.9 ± 3.2 |
| +5% NaCl | 85.7 ± 3.2 | 82.9 ± 2.8 | 50.0 ± 3.8 | 70.4 ± 0.7 |
| + 5 mM H_2_O_2_ | 61.0 ± 5.1 | 85.1 ± 1.2 | 44.1 ± 3.9 | 62.5 ± 3.3 |
| Culture at 20°C | 98.9 ± 0.7 | 85.9 ± 1.8 | 64.9 ± 2.2 | 82.6 ± 1.9 |
| White light irradiation | 98.8 ± 0.5 | 82.7 ± 3.6 | 60.7 ± 2.8 | 80.4 ± 2.6 |

**Figure S1.** Yield of *Rhodotorula glutinis* biomass during the cultivation in control and experimental conditions (a, b, c…—indexes mean homogeneous groups, *—no significant differences, Tukey’s test, α = 0.05)

**Figure S2.** Yield of *Rhodotorula mucilaginosa* biomass during the cultivation in control and experimental conditions (a, b, c…—indexes mean homogeneous groups, *—no significant differences, Tukey’s test, α = 0.05)

**Figure S3.** Yield of *Rhodotorula gracilis* biomass during the cultivation in control and experimental conditions (a, b, c…—indexes mean homogeneous groups, *—no significant differences, Tukey’s test, α = 0.05)

**Figure S4.** Total percentage content of fatty acids synthesized by *Rhodotorula glutinis* yeast after 120 h of cultivation in control and experimental conditions (SFA—saturated fatty acid, MUFA—monounsaturated fatty acids, PUFA—polyunsaturated fatty acids)

**Figure S5.** Total percentage content of fatty acids synthesized by *Rhodotorula mucilaginosa* yeast after 120 h of cultivation in control and experimental conditions (SFA—saturated fatty acid, MUFA—monounsaturated fatty acids, PUFA—polyunsaturated fatty acids)

**Figure S6.** Total percentage content of fatty acids synthesized by *Rhodotorula gracilis* yeast after 120 h of cultivation in control and experimental conditions (SFA—saturated fatty acid, MUFA—monounsaturated fatty acids, PUFA—polyunsaturated fatty acids)
